# Sex Differences in Stroke Recovery

**Published:** 2005-06-15

**Authors:** Lai Sue-Min, Pamela W Duncan, Paul Dew, John Keighley

**Affiliations:** Department of Preventive Medicine and Public Health, The University of Kansas Medical Center; University of Florida, Gainesville, Fla, and Career Research Scientist, North Florida/South Georgia Veterans Health System, Department of Veterans Affairs, Gainesville, Fla; Department of Preventive Medicine, Kansas City University of Medicine and Biosciences, Kansas City, Mo; Department of Preventive Medicine and Public Health, The University of Kansas Medical Center, Kansas City, Kan

## Abstract

**Introduction:**

This study examined differences between men and women in the ability to perform basic activities of daily living, instrumental activities of daily living, and higher physical functioning after stroke. The objective of the study was to determine whether sex differences in stroke recovery can be explained by depressive status beyond older age, stroke severity, prestroke physical functioning, and other medical comorbidities.

**Methods:**

A total of 459 stroke patients were recruited from acute and subacute facilities in an urban midwestern community. These patients were followed prospectively from stroke onset until 6 months poststroke. All study participants were assessed using standardized stroke outcome measures, including the National Institutes of Health Stroke Scale, the Barthel Activities of Daily Living Index, the Lawton Instrumental Activities of Daily Living scale, and the SF-36 Health Survey physical functioning scale. The Geriatric Depression Scale was used to assess depressive status. Each outcome was measured at baseline (within 2 weeks of stroke onset), as well as 1, 3, and 6 months poststroke. Prestroke physical functioning, stroke characteristics, and comorbidities were also assessed at baseline.

**Results:**

Female patients in the study were older than male patients, with a mean age of 71 years for women vs 69 years for men. Female patients reported lower prestroke physical functioning than their male counterparts. Six months after stroke, women in the study were less likely than the men to achieve a score of ?95 on the Barthel Activities of Daily Living Index (hazards ratio [HR] = 0.68; 95% confidence interval [CI], 0.52–0.90), carry out eight of nine instrumental activities of daily living without assistance (HR = 0.46; 95% CI, 0.30–0.68), and score ?90 on the SF-36 Health Survey physical functioning scale (HR = 0.54; 95% CI, 0.28–1.01). When age, prestroke physical functioning, stroke severity, and depressive status at baseline were controlled in the analysis, women in the study continued to be less likely (HR = 0.51; 95% CI, 0.32–0.79) than men in the study to be able to carry out eight of nine instrumental activities of daily living completely without assistance, but there were no observed sex differences in achievement of independence in basic activities of daily living or higher physical functioning.

**Conclusion:**

Prestroke physical functioning and depressive symptoms are important factors in the investigation of sex differences in stroke recovery. Lower recovery of activities of daily living and physical functioning in women after stroke may be due to multifactorial effects of older age, poor physical function prior to stroke onset, and depressive status after stroke.

## Introduction

Reports related to sex differences in functional outcomes after stroke are limited. In one study, women with stroke fared worse than men with stroke on the Camden test, a scale of 16 questions used to assess mental ability relating to orientation and awareness of current affairs (1). Sheikh et al (2) have reported that female stroke patients have more disabilities on discharge than male stroke patients, and female patients have achieved lower scores in activities of daily living (ADL) after stroke (3,4). In addition, compared with male subjects, female stroke patients have been found to be older and to have had more severe deficits in arm function (1-4). A recent report involving 4499 patients from seven European countries found that, after controlling for baseline and clinical factors, female sex was a significant predictor of 3-month disability and handicap following stroke (5). In some investigations, however, the sex differences in outcomes after stroke were inconclusive (6,7). Most of these reports were based on a small number of patients or limited to patients admitted to a rehabilitation unit.

Prestroke physical function and depression have been found to predict stroke recovery (8-13). To date, only the European study has accounted for low prestroke physical functioning in explaining sex differences in disabilities 3 months after stroke. That study reported that women have more disabilities than men 3 months after a stroke even after controlling for prestroke physical functioning (7); however, further investigation into sex differences in stroke recovery is necessary. Depression is also an important factor in stroke recovery (9-11). Several studies have found that depression is more common among female stroke patients (12,13) than male, indicating that depression may also play a role in sex differences in stroke recovery.

This study examined whether sex differences in stroke recovery can be explained by depressive status beyond older age, stroke severity, prestroke physical functioning, and other medical comorbidities. We assessed stroke recovery not only in terms of basic ADL but also instrumental ADL and higher physical functioning.

## Methods

### Study participants

A total of 459 patients who participated in the Kansas City Stroke Study were included in the analysis. This study was reviewed and approved by the University of Kansas Institutional Review Board (IRB). IRB approvals were also obtained from each participating hospital. Patients were enrolled after giving informed consent to participate. Case ascertainment for the Kansas City Stroke Study started in August 1995 and ended in September 1998. Detailed eligibility criteria and recruitment methods have been published elsewhere (14)A total of 459 patients who participated in the Kansas City Stroke Study were included in the analysis. This study was reviewed and approved by the University of Kansas Institutional Review Board (IRB). IRB approvals were also obtained from each participating hospital. Patients were enrolled after giving informed consent to participate. Case ascertainment for the Kansas City Stroke Study started in August 1995 and ended in September 1998. Detailed eligibility criteria and recruitment methods have been published elsewhere (14). Briefly, the eligible study participants were systematically recruited from 12 participating hospitals in the greater Kansas City area. Eligible stroke patients were identified by 1) a review of daily admission records; 2) referrals from physicians, clinical nurse specialists, or therapists in medical, neurology, and rehabilitation units; or 3) review of discharge codes. To be accepted into this study, the subject had to have had a confirmed eligible stroke as defined by World Health Organization (WHO) criteria. WHO criteria define a stroke as "rapidly developed clinical signs of focal (or global) disturbance of cerebral function, lasting more than 24 hours or leading to death, with no apparent cause other than a vascular origin" (15). In this study, strokes were confirmed by clinical assessment and/or by computed tomography or magnetic resonance imaging. Trained nurses or physical therapists reviewed medical records and interviewed both patients and physicians to determine if the patients were eligible and if they consented to enrollment. Informed consent was obtained from all participants and/or proxies.

### Assessment measures

The patients were evaluated using a variety of standardized assessments at enrollment and followed at 1, 3, and 6 months poststroke by a study nurse or physical therapist at the patient's home or at a chronic care facility. Baseline demographics and medical comorbidities were recorded for each participant. The standardized assessment measures used in this study were the National Institutes of Health Stroke Scale (NIHSS) (16), the Geriatric Depression Scale (GDS) (17), the Barthel ADL Index (Barthel Index) (18), the Lawton Instrumental ADL (IADL) scale (19), and the SF-36 Health Survey physical functioning (SF-36 PF) scale (8). The study analyzed baseline NIHSS, baseline GDS, and the 1-, 3-, and 6-month assessments from the Barthel Index, Lawton IADL scale, and SF-36 PF scale. Each study nurse or physical therapist received at least 2 weeks of training in the administration of the assessment measures.

Medical comorbidities of each participant were documented based on chart review as presence or absence of a condition. All study nurses and physical therapists received certification in administration of the NIHSS (16). The NIHSS is a 13-item assessment of neurological function that includes level of consciousness, language, inattention (or neglect), visual-field loss, extraocular movements, motor strength, ataxia, dysarthria, and sensory loss. The NIHSS has scores ranging from 0 to 42, with 42 being fully impaired. Strokes can be further categorized as minor (NIHSS 0–5 points), moderate (NIHSS 6–13 points), and major (NIHSS ≥14 points). Self-reported assessment of patients' physical function 1 week prior to stroke was measured using the SF-36 PF scale.

Symptoms of depression were assessed using the GDS. The GDS has been shown to be a good screening instrument for depressive symptoms and has been tested in stroke patients (20,21). The GDS includes 15 yes/no questions with one point for each depressive symptom. The total score for the GDS ranges from 0 to 15, with 15 being most depressed. A score of ≥6 points can be considered as indicating depression (22-24). The GDS was not scored for patients who were aphasic, who were cognitively impaired as defined by a score of ≤17 points on the Folstein Mini-Mental State examination (25), or who had had a tracheostomy.

The Barthel Index was used to measure basic ADL. The Barthel Index is a weighted instrument that includes a total of 10 items for assessing self care (feeding, bathing, grooming, dressing, toilet use, and bowel and bladder control) and mobility (transfers, ambulation, and stair climbing). It has scores ranging from 0 to 100, with 100 being fully independent in basic ADL. The Lawton IADL scale assesses nine ADLs, such as preparing one's own meals, using the telephone, and taking medications. The patient's ability to do each activity was reported as one of the following: able to do the activity without help, able to do the activity with some help, or completely unable to do the activity. The Lawton IADL scale has a total score ranging from 9 to 27, with 27 being able to independently perform all nine activities.

The SF-36 Health Survey measures eight domains (physical function, limitations in usual role activities because of physical health problems, bodily pain, general health perceptions, vitality, social function, mental health, and limitations in usual role activities because of emotional problems). All domains have a total score ranging from 0 to 100, with 100 indicating the best health. We used only the questions in the physical function domain.

### Statistical analyses

Descriptive statistics were used to show demographics, risk factors, comorbidities, prestroke functional status, stroke characteristics, severity of impairment caused by stroke, and disability and health-related quality-of-life measures. The chi-square test and *t* test were used when appropriate. Survival analysis was done using the Cox proportional hazards regression model (26) to examine sex differences in achieving favorable outcomes. The favorable outcomes were defined as basic ADL independence (Barthel Index ≥95), completion of the nine IADL without assistance, and a score ≥90 on the SF-36 PF scale. As a separate analysis, achieving 90% of prestroke physical functioning, as measured by the SF-36 PF scale, was also examined using the Cox proportional hazards regression model. This model was chosen so that patients who died or were lost to follow-up before achieving favorable outcomes were accounted for and censored at the time of their last participation. The Cox regression also accounted for potential confounding due to older age, stroke severity (NIHSS), depressive symptoms (GDS), prestroke physical functioning, and medical comorbidities. Medical comorbidities were individually included in the regression model as present or absent only when conditions were shown to affect men and women disproportionately in the univariate analysis. To account for all patients, including those whose GDS was not scored at baseline, the Cox proportional hazards regression was analyzed separately by including an indicator variable for those with unscored GDS in the regression model.

## Results

This study included 459 patients who met the Kansas City Stroke Study's eligibility criteria and signed informed consent for participation. Table 1 presents demographics, characteristics, and medical conditions of the study cohort. There were 245 women (53%) and 214 men (47%), 366 whites (80%), 78 blacks (17%) and 15 other race/ethnicity (3%). Female patients were slightly older (*P* = .01) and were more likely to live alone (*P* < .001), to be unmarried (*P* < .001), to be eligible for Medicaid (*P* = .001), and to have had a history of atrial fibrillation (*P* < .001). Male patients, on the other hand, were more likely to be previous smokers (*P* < .001), to be daily alcohol drinkers (*P* = .04), and to have had a history of myocardial infarction (*P* = .001). Medical conditions (other than history of myocardial infarction and atrial fibrillation) and medication use (baby and regular aspirins, Coumadin, and antihypertensives) were similar between the two sexes.

There were other similarities and differences between men and women (data not shown). Self-reported physical function one week prior to stroke was lower among women in the study than it was among men (mean score of 64 ± 29.5 for women on the SF-36 PF scale compared with 76 ± 24.6 for men [*P* < .001]). Mean number of depressive symptoms was similar for women (5 ± 3.0) and men (4 ± 3.0). The percentage of women and men being depressed at baseline was also similar, with 35% of female patients and 30% of male patients scoring ≥6 on the GDS.

Of the 459 stroke cases, 430 (93.7%) were cerebral infarction, and 29 (6.3%) were intracerebral hemorrhages (Table 2). Based on the NIHSS, 237 strokes (51.6%) were categorized as minor, 168 strokes (36.6%) as moderate, and 54 strokes (11.8%) as major. There were no significant differences between men and women in stroke severity, location, type, or symptoms.

Six patients died before the 1-month assessment, 10 subjects declined a follow-up, and three patients moved away. Rates of subject attrition were similar between men and women. Patients who did not complete at least one follow-up were significantly older (77 ± 11.0 years vs 70 ± 11.3 years), had more severe strokes (21% vs 11%), and had more unscored GDS (26% vs 13%). The racial distribution and cognitive function of the completers and noncompleters were similar. Most importantly, female and male noncompleters had similar characteristics, except that the female noncompleters were older, and male noncompleters had more severe strokes.

Univariate analysis showed that female stroke patients were less likely than male stroke patients to achieve basic ADL independence (Barthel Index of ≥95) 6 months after stroke (hazards ratio [HR] = 0.68; 95% confidence interval [CI], 0.52–0.90). Furthermore, women with stroke were less likely than the men to achieve complete independence in eight of nine IADL after 6 months (HR = 0.46; 95% CI, 0.30–0.68). For example, at 3 months poststroke, only 13% of female patients were able to perform eight of the nine IADL completely without assistance, whereas 28% of male patients were able to do so. By 6 months poststroke, 18% of the women could perform these activities, but 34% of the men could. Finally, the women in the study were less likely to score ≥90 on the SF-36 PF scale (HR = 0.54; 95% CI, 0.28–1.01) 6 months after stroke.

Medical comorbidites, including myocardial infarction and atrial fibrillation, had no significant effect on any of the four study outcomes in the Cox regression analysis and were subsequently removed from the final Cox regression modeling. Also, the indicator variable that was intended to capture the effect of not having a GDS score on achieving study outcomes was also excluded from the final HR estimation because of statistical insignificance. The 95% CI for the parameter estimating "unscored GDS" was wide because of a small number of subjects in that group.

Table 3 presents the hazards ratio associated with female sex in achieving the same three favorable poststroke outcomes as men when age, prestroke physical functioning, stroke severity (NIHSS), and depressive status at baseline were controlled for in the analysis. In this more robust analysis, women in the study were still less likely (HR = 0.51; 95% CI, 0.32–0.79) than men to be able to carry out eight of nine IADL without assistance, but there was no sex difference in scoring ≥95 on the Barthel Index or in scoring ≥90 points on the SF-36 PF scale. There was also no sex difference observed in achieving 90% of prestroke physical functioning measured by the SF-36 PF scale when these confounders were considered, as shown in the hazards ratio in Table 4.

## Discussion

Women with stroke were less likely than men to achieve complete independence in basic ADL, IADL, and higher physical functioning. Our study showed, however, that when prestroke physical functioning and depressive status were controlled for in the analysis, there was no difference between the two sexes in achieving independence in basic ADL and higher physical functioning 6 months after stroke. Sex difference remained, however, in independence in IADL.

Our study findings are different from findings observed previously because we included more stroke patients in the study and considered important risk factors for stroke recovery, such as prestroke physical functioning and depression. When sex was the only factor considered, female stroke patients were reported by our study and others to have more disabilities (as measured by the Barthel Index) (1-4,27,28). Only the European multinational hospital-based study considered prestroke physical functioning in its examination of sex differences in stroke recovery. Although the European multinational study considered prestroke functioning, it did not take into account the potential confounding effect of depression and male or female sex on stroke recovery.

One explanation for the persistent difference between men and women in IADL poststroke may be the instrument itself. Many of the activities used in that scale (e.g., preparing meals, shopping for groceries, doing housework) have traditionally been performed by women in the home. Men's traditional role (working outside of the home) may prompt male respondents to report that they were able to do the IADL without help because they typically did not perform these activities either before or after stroke, making a prestroke vs poststroke assessment difficult.

Prevalence of medical comorbidities (e.g., hypertension, diabetes, atrial fibrillation, myocardial infarction) was different between the two sexes, but these comorbidities were found to have no effect on stroke recovery. Previous studies, including two of ours, were also not able to establish consistent patterns between medical comorbidities and stroke recoveries (7,14,29,30). The first of our studies analyzed the medical comorbidities using a composite weighted score of 19 medical conditions (14), and the second study (30) categorized the 19 medical conditions into eight different comorbid domains (cardiac domain, respiratory domain, diabetes, neurological domain, cancer, vision, musculoskeletal domain, and generalized symptoms). In our studies, medical conditions were self-reported in combination with chart review. Some important conditions that are asymptomatic may be underrecognized. Also, disease severity, which was not captured either by self-report or chart review, may explain the inability to establish a relationship between medical comorbidities and stroke recoveries. Future studies examining the relationship among severity of comorbidities, management of comorbidities, and stroke recoveries may be necessary to provide a comprehensive understanding of their relationship. Finally, stroke type (cerebral infarction or intracerebral hemorrhage) was similar between men and women in our study and was shown to have no effect in predicting stroke recovery.

Although our study confirmed that sex differences in stroke recovery affect only independence in performing IADL, we were able to quantify the significant confounding effects of stroke severity, depressive symptoms, and prestroke physical functioning that help explain sex differences in stroke recovery. Our findings have significant public health implications. Although older age is a nonmodifiable risk factor for strokes (31), lower physical functioning in women than men (a well-documented public health issue) can certainly be a target for intervention. The improvement of women's physical functioning is not only significant for eliminating gaps between men and women in stroke recovery but is also an important national objective for healthy elders. A low physical functioning not only affected stroke recovery but also decreased quality of life. Data from the National Center for Health Statistics show that 80% of people older than 65 years have one or more of nine common chronic conditions (including stroke) or impairments (32). A second finding involves individuals who do not have a disease condition but who are nonetheless disabled in some way. In recent years, physical activity, which is considered a viable public health intervention, has been shown to improve stroke recovery and increase an individual's quality of life. The next step would be to develop a medical consensus on modality, dose (frequency and intensity), duration of physical activity, and strategies to increase it (33). 

Although depressive symptoms have been shown to influence stroke recovery adversely, none of the previous studies has taken depressive symptoms into consideration when examining sex differences in disabilities after stroke (9-11,34-37). Our previous study (14) showed that depressive symptoms (either present at baseline immediately after stroke onset or present during follow-up observation) are an obstacle in stroke recovery. Depressive symptoms are common following stroke. Although effective pharmacological interventions are available, depression is inadequately treated (38). Compliance with antidepressant treatment is poor (with compliance rates ranging from 20% to 59%) (38,39). In recent years, exercise has been explored as an alternative method for reducing symptoms of depression. The beneficial effect of exercise on reducing symptoms of depression remains inconclusive, however, because of limitations in study methodologies, design, and size of study populations. A well-designed research study to address issues about exercise and depression is warranted.

We systematically recruited patients from 12 hospitals in academic, community, acute, and subacute care settings in a midwestern community. Our patients' self-reported physical functioning 1 week prior to stroke onset was similar to the national norms for men and women reported by Ware et al (8). Our study results are generalizable to stroke patients who share our recruitment criteria, such as patients who have lived independently in their communities before stroke onset and do not have severe heart conditions.

In summary, the differences in stroke recovery between female and male stroke survivors may be attributed to older age and lower prestroke physical functioning in women. Depression, which may not explain the sex differences in stroke recovery, also modified the recovery patterns. Although old age, sex, and stroke severity are not modifiable, lower prestroke physical functioning and depression *are* modifiable and should be targeted for intervention. Increased physical activity and exercise can improve physical well-being and functioning and may also curtail depressive symptoms. Subsequently, stroke recovery may be more attainable.

## Figures and Tables

**Table 1:** Demographics, Characteristics, and Medical Conditions of Stroke Patients (N = 459) by Sex, Kansas City Stroke Study, 1995–1998

**Characteristic**	**Women (n=245)**	**Men (n=214)**	***P***
**Age, years** **(±SD)**	71 (±12.0)	69 (±10.6)	.01

	**No. (%)**	**No. (%)**	

**Race/ethnicity**			

White	196 (80.0)	170 (79.4)	.55
Black	43 (17.6)	35 (16.4)	
Other	6 (2.5)	9(4.2)	
**Live alone**	97 (39.6)	30 (14.0)	<.001
**Not married**	150 (61.5)^a ^	51 (23.8)	<.001

**Education**			

≤12 years	182 (75.2)^b^	158 (74.2)^b^	.37
College	50 (20.7)^b^	40 (18.8)^b^	
Postgraduate	10 (4.1)	15 (7.0)	

**Insurance status**			

Medicare eligible	189 (77.1)	149 (69.6)	.07
Private insurance	180 (73.5)	167 (78.0)	.54
Medicaid eligible	24 (9.8)	5 (2.3)	.001
**Daily alcohol consumption**	20 (8.2)	30 (14.0)	.04
**Current smoker**	47 (19.2)	53 (24.8)	.15
**Previous smoker**	75 (30.6)	124 (57.9)	<.001

**Medical conditions**			

Diabetes mellitus	75 (30.6)	84 (39.3)	.05
Hypertension (taking medication)	172 (70.2)	142 (66.4)	.37
History of myocardial infarction	31 (12.7)	52 (24.3)	.001
Atrial fibrillation	42 (17.1)	20 (9.3)	<.001
Congestive heart failure	25 (10.2)	18 (8.4)	.51
Chronic obstructive pulmonary disease	28 (11.4)	22 (10.3)	.69
Peripheral vascular disease	35 (14.3)	41 (19.2)	.16
Carotid stenosis	177 (72.2)	149 (69.6)	.54
Previous transient ischemic attack	40 (16.3)	45 (21.0)	.19
Hypercholesterolemia	105 (42.9)	86 (40.2)	.56

a Missing data in one case.

b Missing data in four cases (three women and one man).

**Table 2:** Stroke Characteristics of Subjects (N = 459) by Sex, Kansas City Stroke Study, 1995–1998

**Stroke Characteristic**	**Women (n=245)**	**Men (n=214)**	***P***
Median NIHSS^a^scores	6	5	

	**No.(%)**	**No.(%)**	** **

**Stroke severity**			

NIHSS 0–5 (minor)	121 (49.4)	116 (54.2)	.49
NIHSS 6–13 (moderate)	92 (37.6)	76 (35.5)	
NIHSS ≥14 (major)	32 (13.1)	22 (10.3)	

**Location of stroke**			

Right hemisphere	117 (47.8)	86 (40.2)	.49
Left hemisphere	110 (44.9)	106 (49.5)	
Brain stem	13 (5.3)	16 (7.5)	
Cerebellum	4 (1.6)	5 (2.3)	
Bilateral	1 (0.4)	1 (0.5)	

**Stroke type**			

Cerebral infarction	231 (94.3)	199 (93.0)	.57
Intracerebral hemorrhage	14 (5.7)	15 (7.0)	

**Stroke symptoms experienced**			

Slurred speech	116 (48.5)^b^	96 (44.9)	.43
Weakness and numbness in legs/arms	179 (74.9)^b^	174 (81.3)	.10
Blurred or decreased vision	38 (15.9)^b^	29 (13.6)	.48
Difficulty walking	133 (55.7)^b^	120 (56.1)	.93
Difficulty talking or understanding	69 (28.9)^b^	59 (27.6)	.76

a NIHSS indicates National Institutes of Health Stroke Scale.

b Missing data in six cases.

**Table 3:** Cox Regression Analysis of Sex Differences in Achieving Favorable Outcomes 6 Months After Stroke, Kansas City Stroke Study, 1995–1998

**Factor**	**Hazards Ratio^a^ (95% Confidence Interval)**	***P***

**Outcome: Barthel Index of basic ADL^b^ ≥95**		

Female sex	0.91 (0.66-1.25)	.55
Age (years)	0.96 (0.95-0.98)	<.001
Prestroke physical function	1.02 (1.01-1.02)	<.001
Stroke severity (NIHSS)	0.75 (0.71-0.79)	<.001
Depressive status	0.60 (0.42-0.86)	.005

**Outcome: Completion of eight of nine IADL^b^ without assistance**		

Female sex	0.51 (0.32-0.79)	.002
Age (years)	0.97 (0.95-0.99)	.001
Prestroke physical function	1.03 (1.02-1.04)	<.001
Stroke severity (NIHSS)	0.76 (0.70-0.83)	<.001
Depressive status	0.58 (0.34-0.99)	.04

**Outcome: SF-36 Health Survey physical functioning scale ≥90**		

Female sex	0.79 (0.40-1.57)	.51
Age (years)	0.97 (0.94-1.00)	.05
Prestroke physical function	1.07 (1.04-1.10)	<.001
Stroke severity (NIHSS)	0.80 (0.70-0.90)	<.001
Depressive status	0.15 (0.03-0.62)	.01

a Estimated with Cox proportional hazards regression while adjusting for other factors in the model. Factors removed from the models due to statistical insignificance are medical comorbidities, including atrial fibrillation and myocardial infarction, and unknown depressive status (i.e., unscored Geriatric Depression Scale). Prestroke physical function and National Institutes of Health Stroke Scale (NIHSS) were treated as continuous variables.

b ADL indicates activities of daily living; IADL, instrumental activities of daily living.

**Table 4:** Cox Regression Analysis of Sex Differences in Achieving 90% of Prestroke Physical Functioning 6 Months After Stroke, Kansas City Stroke Study, 1995–1998

**Factor**	**Hazards Ratio^a ^(95% Confidence Interval)**	***P***
Female sex	0.96 (0.40-1.57)	.82
Age (years)	0.97 (0.95-0.98)	<.001
Prestroke physical function	0.97 (0.97-0.98)	<.001
Stroke severity (NIHSS)	0.82 (0.78-0.87)	<.001
Depressive status	0.42 (0.27-0.67)	<.001

a Estimated with Cox proportional hazards regression while adjusting for other factors in the model. Factors removed from the models because of statistical insignificance are medical comorbidities, including atrial fibrillation and myocardial infarction, and unknown depressive status (i.e., unscored Geriatric Depression Scale). Prestroke physical function and National Institutes of Health Stroke Scale (NIHSS) were treated as continuous variables.
